# Congenital Critical Heart Defect Screening in a Health Area of the Community of Valencia (Spain): A Prospective Observational Study

**DOI:** 10.3390/ijns4010003

**Published:** 2018-01-05

**Authors:** Elena Cubells, Begoña Torres, Antonio Nuñez-Ramiro, Manuel Sánchez-Luna, Isabel Izquierdo, Máximo Vento

**Affiliations:** 1 Division of Neonatology, Hospital Universitario y Politécnico La Fe, 46026 Valencia, Spain; 2 Instituto de Investigación Sanitaria La Fe, 46026 Valencia, Spain; 3 Division of Neonatology, Hospital Universitario Gregorio Marañón, 28007 Madrid, Spain

**Keywords:** oxygen saturation, pulse oximetry, critical congenital heart disease, screening, newborn

## Abstract

Despite the progress in the fetal echocardiographic detection of congenital critical heart defects and neonatal physical examination, a significant number of newborn infants are discharged and readmitted to the hospital in severe condition due to cardiac failure or collapse. The aim of this study was to assess the incidence of undetected critical congenital heart disease (CCHD) by a pulse oximetry-screening program in the maternity wards of hospitals with Perinatal Services in a specific geographic area. This is a prospective observational study performed in in the health area corresponding to the city of Valencia. Eligible infants were consecutively admitted newborn infants in the maternities of the participating hospitals with negative fetal echocardiography after normal physical examination in the delivery room. All patients were screened following a specific pulse oximetry protocol before discharge. A total of 8856 newborn infants were screened. A total of three babies presented with severe congenital cardiac malformation and two babies presented with early onset sepsis. Sensitivity was 100% and specificity was 99.97%, with a positive predictive value of 60% and negative predictive value of 100%. Pulse oximetry screening programs in the early neonatal period constitute a valuable tool to avoid inadvertent hospital discharge of severe cardiac malformations and the subsequent life-threatening complications derived.

## 1. Introduction

Congenital critical heart defects (CCHD), defined as those needing invasive medical intervention or those that can produce death within the first 30 days after delivery [1], may in many cases exhibit signs and symptoms that develop after hospital discharge, potentially resulting in collapse and death. Today, prenatal fetal heart ultrasound can detect many such cases, but still some of them can be missed [2]. In addition, both physical examination of the neonate after birth can further detect congenital cardiac malformations, but not all are found [3,4]. Moreover, low oxygen saturation can also be missed clinically [5,6]. Under these circumstances, pulse oximetry appears to be a reliable screening technique in neonates before hospital discharge. It is simple, noninvasive, low-cost, and very reliable in the detection of hypoxemia, and therefore has been recommended as a screening tool [7,8]. Upon adding neonatal pulse oximetry screening to prenatal ultrasound detection and postnatal clinical exam, the diagnosis rate of CCHD increases [9] and thus the undiagnosed cases of CCHD are reduced to less than 10% of total CCHD [10].

In September 2011, the US Health and Human Services (HHS) Secretary’s Advisory Committee on Heritable Disorders in Newborns and Children recommended that critical congenital heart diseases (CCHD) to be added to the neonatal screening panel based on the evidence of a large number of newborns screened in Sweden and England [11,12,13].

In a large meta-analysis in 2012, 13 eligible studies with data for 229,421 newborn babies were analyzed. Pulse oximetry was demonstrated to be highly specific for the detection of critical congenital heart defects with moderate sensitivity [14]. More recently, in a Chinese multicenter study, 122,738 consecutive newborn babies (120,707 asymptomatic and 2031 symptomatic) were screened, detecting congenital heart disease in 1071 (157 critical and 330 major) [15].

We aimed to assess the relevance of universal screening for critical congenital heart disease (CCHD) in a specific health area (Valencia, Spain) where fetal detection of cardiac malformations by echocardiography had been substantially implemented in recent years.

## 2. Population and Methods

This is a prospective observational pilot study performed in the health area of Valencia (Spain). The study protocol was approved by the Internal Review Board (IRB) of the Hospital Universitario y Politécnico La Fe (Valencia, Spain). Parents/guardians of all recruited patients gave informed consent. A total of 12 hospitals, including level I (rural hospital, n = 5), level II (medium sized city hospital, n = 5), and level III hospitals [2], were included for a 6-month period. Eligible infants were newborn babies admitted to the maternity wards with normal fetal ultrasound evaluation for CCDH and normal newborn examination by a neonatologist in the delivery room. Babies with suspicious fetal echocardiography or abnormalities in the physical examination (color, murmurs, pulse abnormalities) were evaluated by a pediatric cardiologist and disregarded for the study.

**Figure 1:**
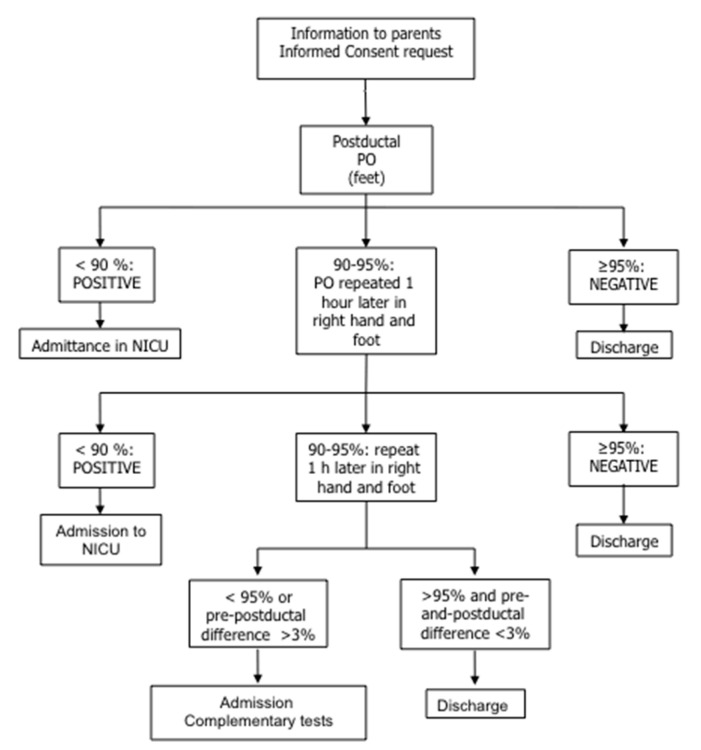
Flow diagram describing the protocol of the Pulsimat study for the screening of critical congenital heart disease. PO: pulse oximetry.

Included patients were screened between 24–48 h after birth, following the protocol shown in Figure 1. Newborn infants with a positive or doubtful screening were admitted to the Neonatal Intensive Care Unit (NICU) where the clinical, analytical, and echocardiographic study was completed by neonatologists and pediatric cardiologists. After reaching a diagnosis, babies remained in the NICU for treatment or were discharged home.

## 3. Results

**Figure 2:**
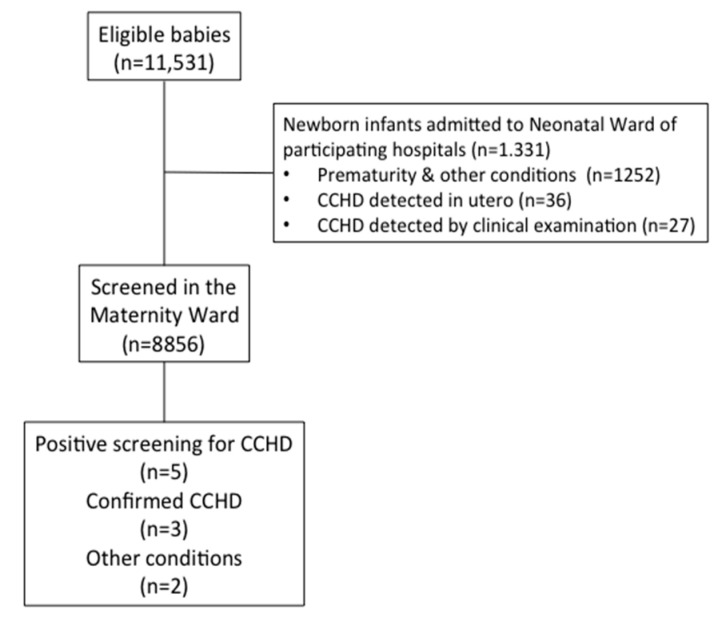
Consort diagram describing eligible patients, disregarded, and finally screened for critical congenital heart disease (CCHD) in the Pulsimat study.

Figure 2 shows the flow diagram of the study. From a total of 11,531 newborn infants born in the participating hospitals, 66 babies were diagnosed with CCHD, representing 5.8 per thousand newborn infants. Out of these, 36 patients had CCHD detected in utero and confirmed at birth (3.1 per 1000 births), 27 were diagnosed after birth based on clinical symptoms and echocardiography (2.3 per 1000 births), and three were detected by pulse oximetry screening at discharge (0.26 per thousand live births). A total of 8856 representing 78.6% of all births were screened for critical CCHD. Out of these, five had a positive pulse oximetry screen. However, two cases were caused by respiratory distress secondary to early onset sepsis; thus, only the remaining three cases were due to CCDH, specifically, total anomalous venous return.

Hence, in the 6-month study period, a total of 66 babies were diagnosed with CCHD; 36 in utero (54.5%), 27 (41.6%) ex utero by clinical examination, and three by pulse oximetry screening at discharge, representing 4.5% of the total instances of CCHD.

Table 1 shows the values for sensitivity, specificity, positive predictive value, and negative predictive value.

## 4. Discussion

It has been estimated that 30% of all CCHD patients have a late diagnosis and could benefit from neonatal screening [16]. As most previous studies demonstrated that non-cardiac conditions could also be detected by pulse oximetry, false positive detection can represent another advantage of the screening [13,17]. In our study, the detection of CCHD in utero (fetal echocardiography) plus ex utero after clinical examination represented 95.5% of the total, while 4.5% of the babies with CCHD were detected by pre-discharge screening. Interestingly, two patients that would have been discharged home with early onset sepsis had a positive screening and were admitted to the NICU. In June 2013, an international group of neonatologists and cardiologists met in Torino (Italy) with the chief investigators from two European screening studies of pulse oximetry to discuss strategies to develop Europe-wide recommendations for CCHD screening. They concluded that there is a need for a European Consensus on CCDH screening in Europe [18]. Recently, a panel from The European Pulse Oximetry Screening Workgroup investigating pulse oximetry screening for critical congenital heart defects published a European consensus statement that recommended the use of pulse oximetry for the early detection of CCDH in all European countries, using new-generation equipment that is motion tolerant; screening after 6 h of delivery and before discharge from the birth center, preferably within the 24 h after birth; and using two extremities, the right hand and either foot [19]. In addition, guidelines for the management of CCDH have been implemented, including practical aspects and nomograms to facilitate the management of these babies [20]. Finally, the Committee on Standards of the Spanish Neonatal Society (SENeo) has very recently published the National Guidelines for the Screening of Critical Congenital Heart Defects [21]. SENeo recommends performing the screening at >24 h after birth; however, each center should analyze its owns circumstances; in some centers performing screening at <12 h after birth could be preferable, despite the increase in false positives. If express discharge is performed, then any time after birth is recommended. It is also recommended to perform pre- and post-ductal pulse oximetry with motion tolerant devices that are also reliable at low saturations. The ranges of normality are similar to those defined by the European Consensus, with an absolute positivity with SpO2 values <90% and negativity with values ≥95% in any extremity, and a difference ≥3% between pre- and post-ductal pulses. In this scenario, we encourage every European country to implement the guideline that best suits their health organization and characteristics of the population.

Our study has obvious limitations. The number of patients and the geographical area are both small, and thus do not represent the entire Valencian community with 55,000 deliveries per year. However, it reveals that despite the excellent prenatal and postnatal detection of CCHD, approximately 5% of these babies would have been discharged home from the hospital. We could speculate that if we translate these numbers to the entire population of our community, at least 15 newborn infants could have been discharged yearly with a life-threatening condition, although the data obtained in our study do not support this conclusion. We did not see any false negative cases, although they have been described in other studies. Unluckily, the number of patients included in our study was relatively small and hindered the possibility of detecting such cases. In addition, our study revealed that it is feasible to systematically perform pulse oximetry screening in the maternity ward without implying a relevant additional workload for the nursing staff. Finally, it is worth mentioning that pulse oximetry screening can also promptly detect other severe conditions, such as early onset sepsis, that went undiagnosed in the routine clinical examination. The Spanish Neonatal Society (SENeo) is very soon going to put forward a protocol for the screening of CCHD that will be adopted by all the maternity wards in our country [21].

## Figures and Tables

**Table 1:** Screening for congenital cardiac heart defects: analysis of results.

Echocardiography			
Pulse oximetry	Positive	Negative	Total
Positive	3	2	5
Negative	0	8851	8851
Total	3	8853	8856
